# Assessing the state of deep-sea biological knowledge and charting a path for scientific research in Barbados

**DOI:** 10.7717/peerj.21380

**Published:** 2026-07-07

**Authors:** Kyle Foster, Muriel Rabone, Juliano Palacios-Abrantes, Judith Gobin, La Daana Kada Kanhai, Henri Vallès, Diva Amon

**Affiliations:** 1 Department of Life Sciences, Faculty of Science and Technology, University of the West Indies St. Augustine, Port of Spain, Trinidad and Tobago; 2 Natural History Museum, London, United Kingdom; 3 Institute for the Oceans and Fisheries, The University of British Columbia, Vancouver, British Columbia, Canada; 4 Department of Biological & Chemical Sciences, University of the West Indies, Cave Hill, Barbados; 5 Marine Science Institute, University of California, Santa Barbara, Santa Barbara, CA, United States of America; 6 SpeSeas, D’Abadie, Trinidad and Tobago

**Keywords:** Biodiversity, Marine scientific research, Baseline, Small Island Developing State (SIDS), Barbados, Roadmap

## Abstract

For Small Island Developing States, including those in the Caribbean, the deep sea remains a challenging ecosystem to study as it requires expensive technology and specialised expertise. The deep-sea environment of Barbados (200–5,776 m) accounts for 99.8% of its Exclusive Economic Zone (EEZ). Barbados is presently in the initial phases of developing a marine spatial plan for its EEZ. Comprehensive baseline information on the deep-sea ecosystems is crucial for evidence-based decision-making. This research assesses the state of deep-sea biological knowledge in Barbados *via* a comprehensive review of species records from peer-reviewed and grey literature, as well as museum and biodiversity databases. We found 1,589 biological records constituting 309 families and 624 species and morphospecies for Barbados’ deep sea. Strikingly, richness analyses estimate that just 20% of the species and 48% of the families inhabiting Barbados’ deep sea have been recorded. We also discuss limitations in existing knowledge, including vast geographic areas and depths still requiring research, as well as the varied methods of deep-sea observation and sampling utilised thus far, and the associated influence on the taxonomic composition of the known deep-sea community. Although limited, this assessment of deep-sea biodiversity information has provided insights to assist with the creation of a road map which decision-makers can utilize to guide future management and research activities.

## Introduction

Barbados is the most easterly island in the Caribbean. It is categorized as a Small Island Developing State (SIDS), with a land area of approximately 432 km^2^ (Roberts, Downes & Simpson, 2020). Its maritime area, however, is around 187,000 km^2^; more than 400 times larger (Roberts, Downes & Simpson, 2020; Humphrey & Alleng, 2021). Approximately 99.8% of Barbados’ Exclusive Economic Zone (EEZ) is classified as deep ocean, *i.e.,* waters which are deeper than 200 m, making it the country with the highest proportion of deep water in the Americas (Hendrickx, 2020; Johannes, 2024). The island’s EEZ reaches depths of 5,766 m (Johnston, McGlynn & Prado, 2020), with most of the deep-sea habitats understudied (Roberts, Downes & Simpson, 2020). This is primarily due to a chronic lack of technology, institutional capacity, and funding, and to a lesser extent, a shortage of expertise in the Caribbean as a whole (Howell et al., 2020; Amon et al., 2022a; Bell et al., 2023; Allcock et al., 2025).

The geology of Barbados’ deep ocean, however, is relatively well known (Speed & Larue, 1982; Ahmed, 2016). The island sits on a large geological structure known as the Barbados Accretionary Prism (BAP) (Speed & Larue, 1982; Ahmed, 2016). Barbados’ origin is largely sedimentary; formed by the off-scraping of sediments deposited from the Orinoco fluvial system on the continent of South America (Faugeres et al., 1990; Hill & Schenk, 2005; Ahmed, 2016). The uplift of this sediment as the South American plate subducts beneath the Caribbean plate has created the 300-km wide and 20-km thick BAP, with the subaerially exposed coralline portion being known as the island of Barbados (Schoonmaker, Mackenzie & Speed, 1986; Donovan & Harper, 2005; Ahmed, 2016). The over-pressurization of the sediment due to depth and sediment loading, among other factors, has led to the development of oil and gas deposits and the expulsion of liquified materials and gases, resulting in the formation of mud volcanoes and methane seeps with associated chemosynthesis-based communities (Faugeres et al., 1990; Jollivet et al., 1990; Olu et al., 1997; Sibuet & Olu, 1998; Deville et al., 2003; Hill & Schenk, 2005).

Barbados’ marine waters support a robust Blue Economy, which currently includes coastal and pelagic fisheries, tourism, shipping, and may expand to include oil and gas extraction and ocean-based renewable energy (Roberts, Downes & Simpson, 2020). Barbados is also in the process of developing a marine spatial plan (MSP) for its EEZ, which aims to protect 30% of the area as a part of the Convention on Biological Diversity’s Global Biodiversity Framework’s Target 3 (Conference of the Parties to the Convention on Biological Diversity, 2022; Wiggins, 2022). As Barbados’ EEZ is predominantly deep sea, data on its pelagic and benthic deep-sea biodiversity are critical for well-informed sustainable management and conservation.

In light of the need for biological baseline information on Barbados’ deep sea, this study sought to synthesize the publicly-available deep-sea biodiversity data collected in Barbados to date, sourced from peer-reviewed and grey literature, online biodiversity databases, and museum databases. This effort intends to provide the Barbados Marine Spatial Plan Unit, and other government agencies, which operate within the ocean space, with unique insights into the existing trends and gaps in deep-sea biological knowledge, as well as inform their path for research and management for Barbados’ deep-sea biodiversity as it undertakes its current MSP process and beyond.

## Materials and Methods

### Data collection

The methodology utilised follows that of Lue Chin et al. (2026). Barbados’ deep-sea biological records were drawn from several sources during 2024. The first step included the biodiversity databases, Ocean Biodiversity Information System (OBIS) and Global Biodiversity Information Facility (GBIF), where the search functions were used to filter records by country. As a second step, sample repositories (*e.g.*, international museums) that were listed in the OBIS and GBIF search results were then searched individually in case not all records had been successfully integrated into OBIS or GBIF. These included the Florida Museum of Natural History’s (UF) Fish and Invertebrate collections, California Academy of Sciences (CAS) Invertebrate and Ichthyology collections, Smithsonian National Museum of Natural History Fishes and Invertebrate collections, and the Muséum National D’histoire Naturelle. Additionally, utilising our local knowledge of where biological samples or records may be hosted in Barbados, records were sought from local institutions and agencies, such as Barbados’ Ministry of Environment and National Beautification, the Centre for Resource Management and Environmental Studies at the University of the West Indies’ Cavehill Campus, the Barbados Museum & Historical Society, and the Bellairs Research Institute of McGill University. However, no records were obtained from these sources.

As a third step, additional records were sourced from peer-reviewed literature. Google Scholar was used to search peer-reviewed articles and grey literature, written in English, that contained the terms “Barbados”, “species” and “deep sea”, while excluding “fossil”. This yielded 5,780 results. Each of these was then examined to ascertain which included deep-sea species records for Barbados. Of these, only ten included biological information on multicellular organisms (Nutting, 1919; Van Soest & Stentoft, 1988; Le Pichon et al., 1990; Guezennec & Fiala-Medioni, 1996; Henry et al., 1996; Vacelet et al., 1996; Olu et al., 1997; Vacelet & Boury-Esnault, 2002; Olu et al., 2010; Toone & Washburn, 2020). Records were also incorporated from accessible cruise reports and sample lists. These reports and lists again stemmed from research cruises listed in the OBIS and GBIF search results, as well as the literature search. However, the only report and sample list successfully located was from RV *Atlantis* in 2012 (Van Dover, 2012). Data generated by the private sector were not publicly available and thus were not included, limiting the evaluation of commercial environmental baseline data, *e.g.*, Environmental Impact Assessments (EIAs). Despite these exhaustive methods, there are likely to be sleeping data/samples—unpublished, unstandardized, or inaccessible, and yet valuable data, particularly when combined with other datasets—that have not been incorporated.

### Data quality assessment and control

Downloaded records were then filtered by depth to include only records 200 m or deeper. Duplicate records were also removed. Records with unresolvable missing critical information, such as depth or coordinates, were also excluded to ensure data accuracy and reliability. The data were also cleaned by location using the R statistical software (R Core Team, 2024) and associated packages *sf* and *sp*, with any records falling outside of Barbados’ EEZ or on land removed. These were identified through point data analysis using a map consisting of Barbados’ EEZ shapefile, provided by Flanders Marine Institute (2023), and bathymetry sourced from General Bathymetric Chart of the Oceans (GEBCO) 2024 Grid.

Additionally, the taxonomic information of each record was reviewed and higher taxonomy updated in accordance with the World Register of Marine Species (WoRMS). Only metazoan biological records were included in the dataset, with records labelled ‘biota’ and ‘paleontological’ deleted. A number of records gathered from the ChEssBase database on OBIS, which are confirmed species endemic to the East Pacific Rise hydrothermal vents, were also excluded on account of inaccuracy.

A unified database structured following the Darwin Core global biodiversity data standard (Wieczorek et al., 2012) was consolidated and is provided in Table S1. Additionally, records not previously included on OBIS have been uploaded: https://obis.org/dataset/e130c58b-3f35-4353-93ec-1b7ad028325f.

### Data analyses

The dataset was compiled from expeditions that employed different sampling methods, with varying objectives, intensities, and spatial coverage, which introduces inherent bias and limits comparability between research expeditions and samples. To reduce these biases, several actions were taken. Records were categorized according to whether they were collected in the water column (pelagic) or on the benthos (benthic). This was primarily determined by the sampling protocols used. Morphospecies collected by the 10′ Isaacs-Kidd Midwater Trawl (IKMWT), rectangular midwater trawl (RMT), 10 sq. m MOCNESS, longline, and the Net - NV 70 were categorized as ‘pelagic’, while those collected by dredge (ashophyton and tumbler), trawl (large beam and shrimp - 40ft), epibenthic sled, Human-Occupied Vehicle (HOV), and Remotely Operated Vehicle (ROV) were categorized as ‘benthic’. For those records without documented sampling protocols, the morphospecies were checked against FishBase for fish species, SeaLifeBase for non-fish species, and/or WoRMS, and/or the peer-reviewed literature to verify their respective environments. Benthopelagic species were treated as benthic, reflecting their association with the seafloor. Additionally, during analyses, each record was treated as reflecting presence/absence and did not include any individual counts nor counts derived from area-based sampling.

Family and species richness were calculated based on the total number of invertebrate and fish families and species identified, respectively. Records identified only to the genus, family, or putative species level were included, provided they represented unique records for Barbados. For example, *Eustomias variabilis* was included but *Eustomias sp.* was not. However, if there was no other record of the genus *Eustomias*, then *Eustomias sp.* was included. To evaluate sampling completeness, rarefaction and extrapolation analyses were performed using the Chao2 incidence-based estimator (sample-based) following Rabone et al. (2023). These analyses were conducted in R using the package iNEXT (Hsieh, Ma & Chao, 2016). Species richness and family richness rarefaction curves were calculated using the vegan R package (Oksanen et al., 2022). Individual species were considered to be one sampling unit, however, this assumed that individual records were considered to be individually obtained. The code to replicate these analyses is available at https://doi.org/10.5281/zenodo.19361030.

## Results

### Locations and depths of records

**Figure 1:**
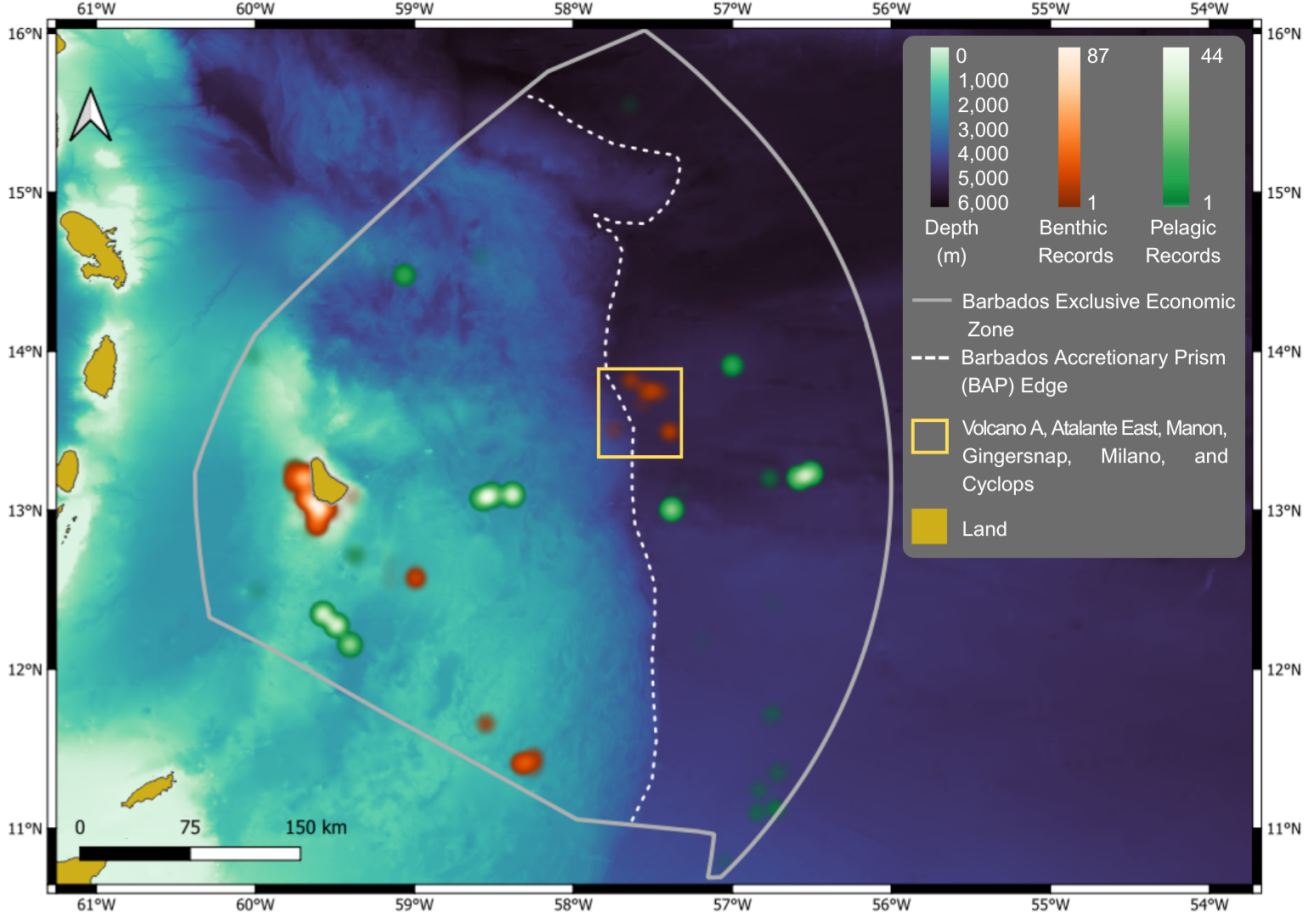
Distribution and depth of benthic and pelagic deep-sea biological samples in Barbados’ Exclusive Economic Zone. EEZ shapefile: Flanders Marine Institute (2023). Maritime Boundaries Geodatabase: Maritime Boundaries and Exclusive Economic Zones (200NM), version 12. Bathymetry: General Bathymetric Chart of the Oceans (GEBCO) 2024 Grid. Caribbean Islands Shapefile: Caribbean Coastline NGAPrototype 2014 by The Nature Conservancy.

This study identified 1,589 biological records from the deep sea within Barbados’ EEZ (Table S1). Of these, 977 were collected from the benthos and 612 from the water column (Table S1). The majority of the benthic records were collected close to shore, predominantly on the south and west coasts, while pelagic records dominated further offshore (Fig. 1). Most records were also concentrated in clusters between 11° and 14^o^ North, whereas there were very few pelagic records and no benthic records more northward (Fig. 1). There were also concentrations of records east of 60° West, the majority of which were pelagic (Fig. 1). There were 876 benthic and 416 pelagic records collected on or above the BAP (55.1% and 26.2% respectively), whereas 101 benthic records and 196 pelagic records (6.4% and 12.3%) were collected elsewhere. Pelagic records collected beyond the BAP were more geographically scattered, whereas benthic records were constrained to a relatively small area of mud volcanoes (Volcano A, Atalante East, Manon, Gingersnap, Milano and Cyclops) on the BAP eastward edge (Fig. 1).

**Figure 2:**
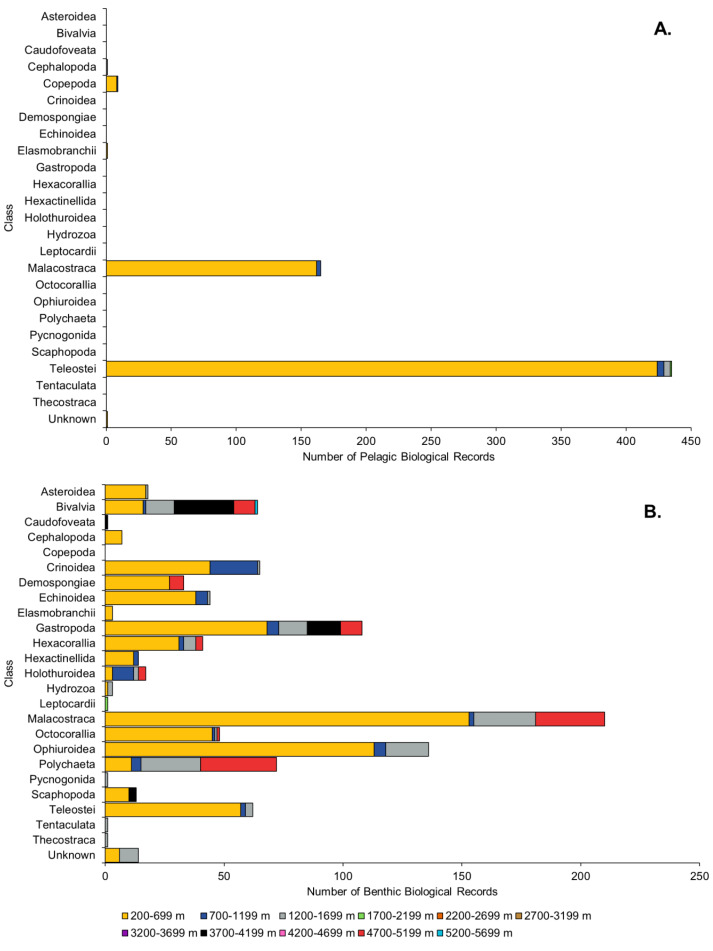
Number of benthic and pelagic biological records by taxonomic composition and depth from Barbados’ deep sea. (A) Pelagic records; (B) Benthic records.

Consequently, an analysis by depth revealed that 79% of the records collected or observed occurred in the shallowest depth category (200–699 m) (Figs. 2A & 2B). This was composed of 824 benthic and 662 pelagic records. The number of records decreased substantially below 699 m depth (Figs. 2A & 2B). Four percent of the records (58 benthic and nine pelagic) were observed from depths of 700–1,199 m. At depths of 1,200–1,699 m, 8% of the records were documented, composed of 120 benthic and six pelagic. At 1,700–2,199 m, 0.1% of the records were documented, with one benthic and one pelagic. No pelagic records were recorded below the 1,700–2,199 m depth class. The depths 3,700–4,199 m held 3% (*n* = 43) of the records, 6% (*n* = 92) at 4,700–5,199 m, and there was only one record (0.06%) between 5,200 and 5,699 m, all benthic. Importantly, no records were documented between 2,200 and 3,699 m, and 4,200 and 4,699 m (Figs. 2A & 2B).

### Taxonomic classification of records

**Figure 3:**
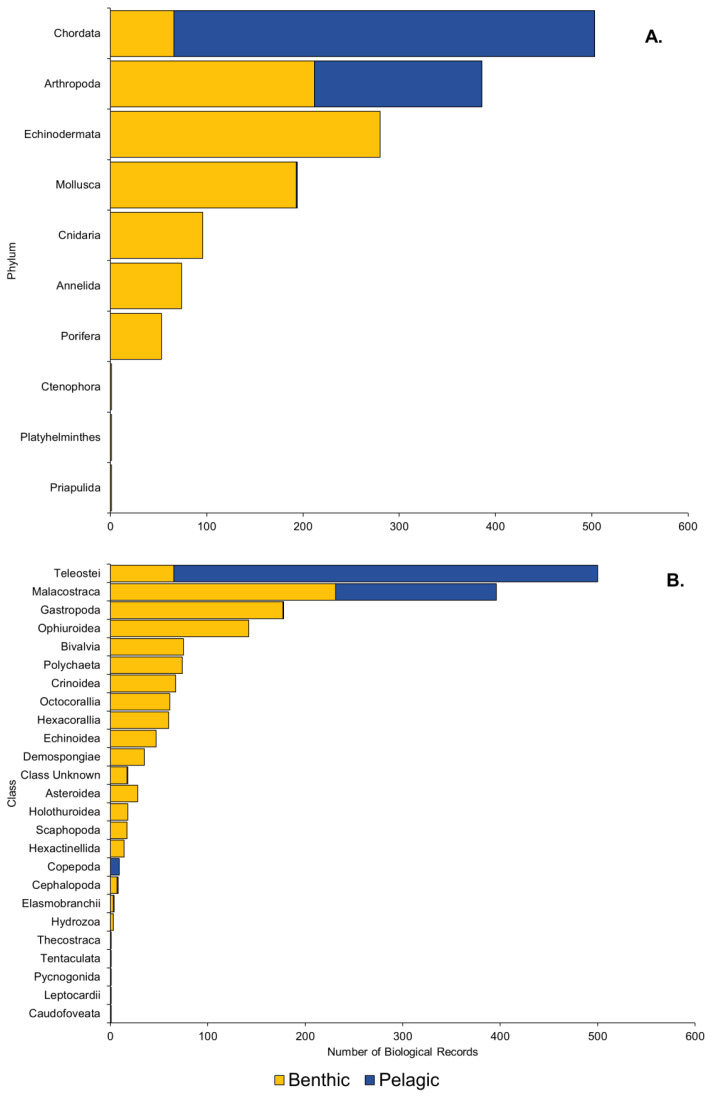
Number of benthic and pelagic records from Barbados’ deep sea. (A) Records by phylum. (B) Records by class.

The 1,589 records represented ten phyla, 24 classes, 109 orders, 309 families, and 624 species and morphospecies (Tables S1 and S2, Fig. 3). Chordata was the most prevalent phylum, comprising approximately 32% of the records (66 benthic and 437 pelagic), while Arthropoda was the second most recorded (24%, 212 benthic and 174 pelagic records) (Fig. 3A). Following those orders were Echinodermata (17%, 280 benthic records), Mollusca (12%, 193 benthic and one pelagic records), Cnidaria (6%, 96 benthic records), Annelida (5%, 74 benthic records) and Porifera (3%, 53 benthic records) (Fig. 3A). Ctenophora, Platyhelminthes, and Priapulida were the least represented with one record each, all benthic (Fig. 3A).

**Figure 4:**
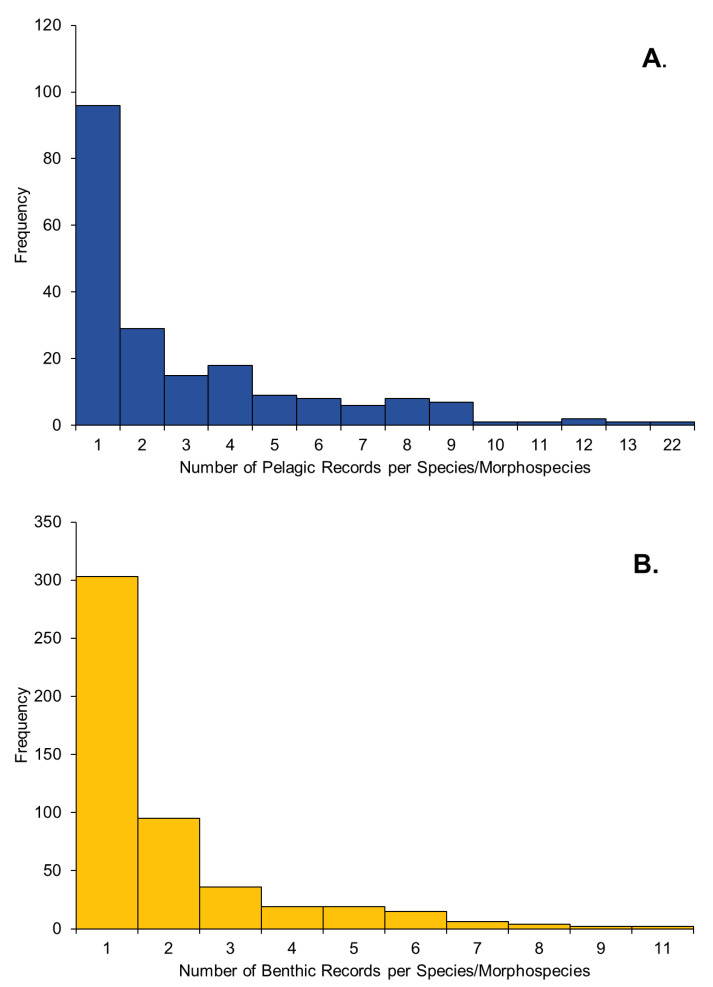
The number of records per (A) pelagic and (B) benthic species/morphospecies from Barbados’ deep sea. There were 96 pelagic and 303 benthic species sampled only once, while *Gobiidae* sp. (22 records), and *Gastropoda* sp. and *Neocrinus blakei* (both 11 records), were the most sampled pelagic and benthic records respectively.

By class, Teleostei was the most recorded, representing 31% of the records (62 benthic and 435 pelagic), followed by Malacostraca at 24% (210 benthic and 165 pelagic records) (Fig. 3B). Therefore, over half of the records consist of only these two classes. Contrastingly, Caudofoveata, Leptocardii, Pycnogonida, Tentaculata, and Thecostraca, all benthic, each only had one record (Figs. 2, 3B). When analyzing classes by depth, 34% of the 1,421 records were teleosts between 200 and 699 m, followed by the class Malacostraca (23%) in the same depth category (Fig. 2). This was also the most diverse depth interval, with 79% (*n* = 19) of the classes recorded in this assessment represented. The 1,200–1,699 m depth interval followed closely behind with 58% (*n* = 17) classes represented at this depth (Fig. 2). At the deepest depth interval (5,200–5,699 m), there was only one record (Bivalvia) (Fig. 2B).

The most recorded morphospecies was *Gobiidae* sp., comprising 1.4% of all the records (*n* = 22) which were all pelagic and collected by 10′ IKMWT during the RV *Atlantis II* cruise in 1973 (Table S1). It is clear that the majority of the species and morphospecies were sampled only once, with 399 (25%) occurring only once. This comprises 303 (76%) benthic, and 96 (24%) pelagic morphospecies (Fig. 4). Of the benthic records, only two morphospecies had more than ten records collected, with both Gastropoda sp. and *Neocrinus blakei* having 11 records respectively. In contrast, six pelagic morphospecies had over ten records: Gobiidae sp. (22 records), *Vinciguerria nimbaria* (13 records), *Gardinerosergia splendens* (12 records), *Thunnus albacares* (12 records), *Gennadas scutatus* (11 records), *Gennadas bouvieri* (ten records). Twenty percent of the species that were recorded only once were from waters deeper than 1,000 m. Overall, the percentage of records classified to species level stood at 23.2%, 85.8% classified to the genus level, 93.5% to the family level, 95.8% to the order level, and 99% identified to class.

Methane-seep inhabiting species at the mud volcanoes previously identified (*e.g.*, *Abyssogena southwarde*, *Gigantidas mauritanicus*, *Lamellibrachia* sp. and *Cladorhiza methanophila*), observed and collected by HOV *Nautile* and ROV *Jason II,* accounted for 12% of the total records (*n* = 189) (Tables S1 and S2). Amphipoda sp., was the most collected species at the mud volcanoes, Volcano A, Atalante East, Manon, Gingersnap, Milano, and Cyclops overall, with 23 records (Table S1; Fig. 1).

### Methods of collection and observation

Publicly available information revealed 21 research expeditions that conducted biological sampling or observation in Barbados’ deep waters between 1871 and 2023 (Table 1). Scientific equipment and associated collection methods evolved over time, gradually shifting from dredging and trawling during the earliest expeditions, to the use of HOVs and ROVs from the 1980s onwards (Table 1). Six expeditions used pelagic sampling methods: RV *Oregon*, RV *Atlantis II* (1964, 1966, 1973), RV *Columbus Iselin*, and RV *Chain*. Nine expeditions employed benthic sampling methods. Of these, five used benthic trawling methods: RV *Oregon*, RV *Albatross*, University of Iowa: Antigua Expedition, RV *Diadema*, and the RV *Knorr,* while three utilized deep submergence vehicles (RV *Le Nadir*, RV *Seward Johnson*, and RV *Atlantis II*). Six expeditions, namely, the RV *James M. Gillis*, MV *Fregata*, Undaunted, Dana expedition, Hassler expedition, and the United States Coast Survey Steamer “Blake” (USCSS *Blake*), did not have sampling methods documented. Additionally, few expeditions utilized gears such as sediment cores, thus existing data are largely biased towards megafauna.

**Table 1:** Expeditions that conducted biological sampling in the deep ocean (>200 m) within Barbados’ Economic Exclusive Zone.

**Expedition name/cruise number**	**Year**	**Collection method**	**Benthic/Pelagic**	% **of total samples** ***n* = 1, 589**
Hassler Expedition	1871	N/A	N/A	0.1
USCSS *Blake*	1879	Dredge	N/A	35.4
RV *Albatross*	1887	Large Beam Trawl	Benthic	1.2
University of Iowa Barbados: Antigua Expedition	1918	Dredge	Benthic	0.3
Dana Expedition	1921	N/A	N/A	0.1
RV *Atlantis II*	1964	Net - NV 70	Pelagic	0.2
RV *Diadema*	1964	Dredge	Benthic	0.6
RV *Oregon*	1964	Dredge, Trawl, Longline	Benthic and Pelagic	3.9
RV *Chain*	1965	Net - NV 70	Pelagic	0.3
Undaunted	1965	N/A	N/A	0.1
RV *Atlantis II*	1966	Isaacs-Kidd Midwater Trawl	Pelagic	2.6
MV *Fregata*	1968	N/A	N/A	0.1
RV *James M. Gillis*	1972	N/A	N/A	0.1
RV *Knorr*	1972	Epibenthic Sled	Benthic	1.6
RV *Atlantis II*	1973	Isaacs-Kidd Midwater Trawl	Pelagic	29.0
RV *Columbus Iselin*	1983	RMT - 10	Pelagic	2.8
RV *Le Nadir*	1987, 1992	HOV *Nautile*	Benthic	0.6
RV *Seward Johnson*	1989	HOV *Johnson Sea Link*, Dredge	Benthic	2.3
RV *Atlantis*	2012	ROV *Jason II*	Benthic	11.3
RRS *James Cook*	2023	ROV *Isis*	Benthic	N/A
Unknown	N/A	N/A	N/A	7.5

**Figure 5:**
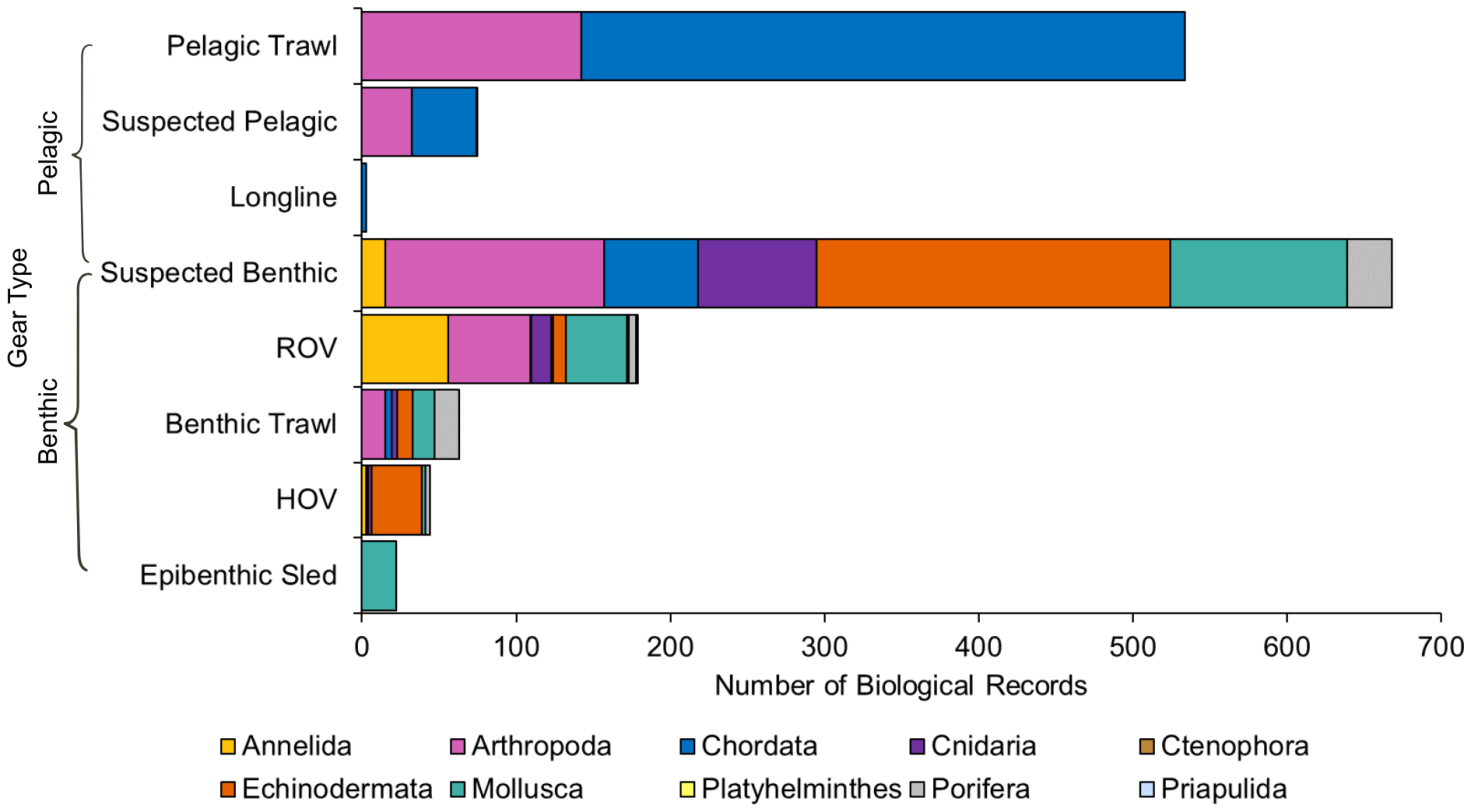
Biological records observed or sampled from Barbados’ deep sea by gear type and phyla. Pelagic sampling gears include the longline and ‘Pelagic Trawl’, which encompasses the Isaacs-Kidd Midwater Trawl, rectangular midwater trawl, 10 sq. m MOCNESS and the Net-NV 70, as well as those samples suspected to be caught using pelagic gears. Benthic sampling gears include the Remotely Operated Vehicle (ROV), Benthic Trawl (all other types of dredging and trawling), Human-Occupied Vehicle (HOV), and Epibenthic Sled. Suspected Pelagic and Suspected Benthic records were missing sampling protocols; however, these species were checked against FishBase and SeaLifeBase to verify whether species were pelagic or benthic.

Pelagic trawling appears to be the most used sampling protocol across expeditions in Barbados, resulting in 34% of total records collected or observed (*n* = 534) (Fig. 5). Within this category, the 10′ IKMWT collected the most samples (*n* = 473). Chordata and Arthropoda were the only two phyla collected by the 10′ IKMWT, with Chordata comprising 339 records and Arthropoda 134 records (Fig. 5). The second highest number of records was by ROV (11%, *n* = 179) and was the only method that sampled all ten phyla observed in Barbados’ deep sea (Fig. 5). Contrastingly, both the epibenthic sled and longline were the methods that recorded the least phyla, with only one each (Fig. 5). The sampling method that collected the least number of records was longline (three records from Chordata) (Fig. 5).

In terms of sampling method and depth, ranges of 200 to 4,199 m saw a diversity of gears utilised, whereas waters deeper than 4,200 m were only sampled by ROVs and HOVs. Of the 1,589 records documented for Barbados, 92% (1,470) were collected specimens (Fig. 5). Of these, 896 were collected from the benthos, and 574 were pelagic.

**Figure 6:**
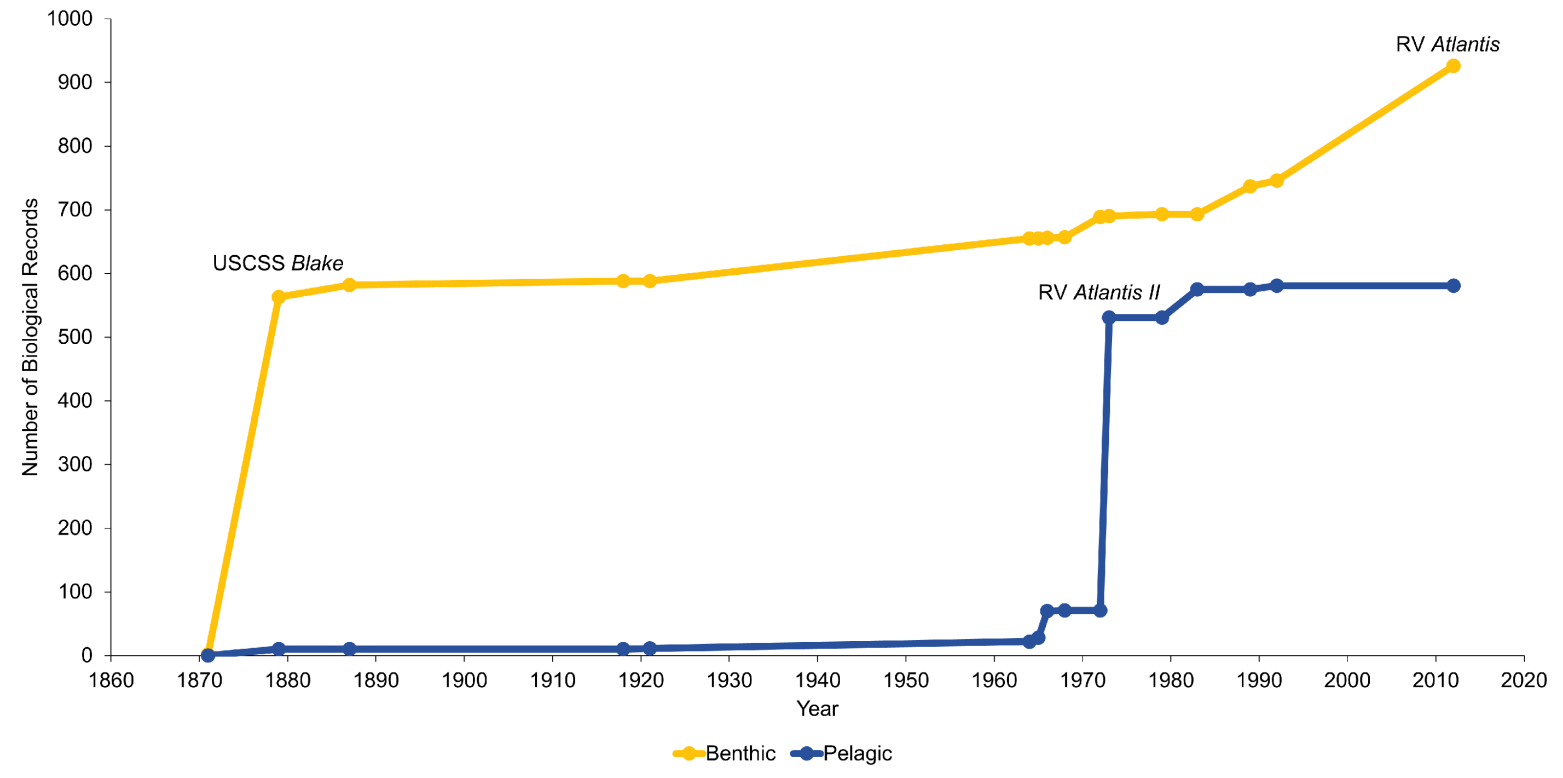
Cumulative deep-sea invertebrate and fish records collected during expeditions from 1871 to 2012 within Barbados (*n* = 1,470 of the total 1,589 records). These are split according to whether they were sampled from the benthos (Benthic) or water column (Pelagic). A total of 119 records were excluded due to missing dates or expeditions. The three expeditions that contributed the highest number of records are labelled.

The number of new biological records collected saw three sharp increases over the years, stemming from the USCSS *Blake* (1879), RV *Atlantis II* (1973), and RV *Atlantis* (2012) (Fig. 6). That is, these expeditions accounted for 35%, 29%, and 11% of the overall records, respectively. The USCSS *Blake* collected 552 benthic and 10 pelagic records, RV *Atlantis II* collected one benthic and 460 pelagic records, and the RV *Atlantis* collected 180 benthic samples (Fig. 6).

**Figure 7:**
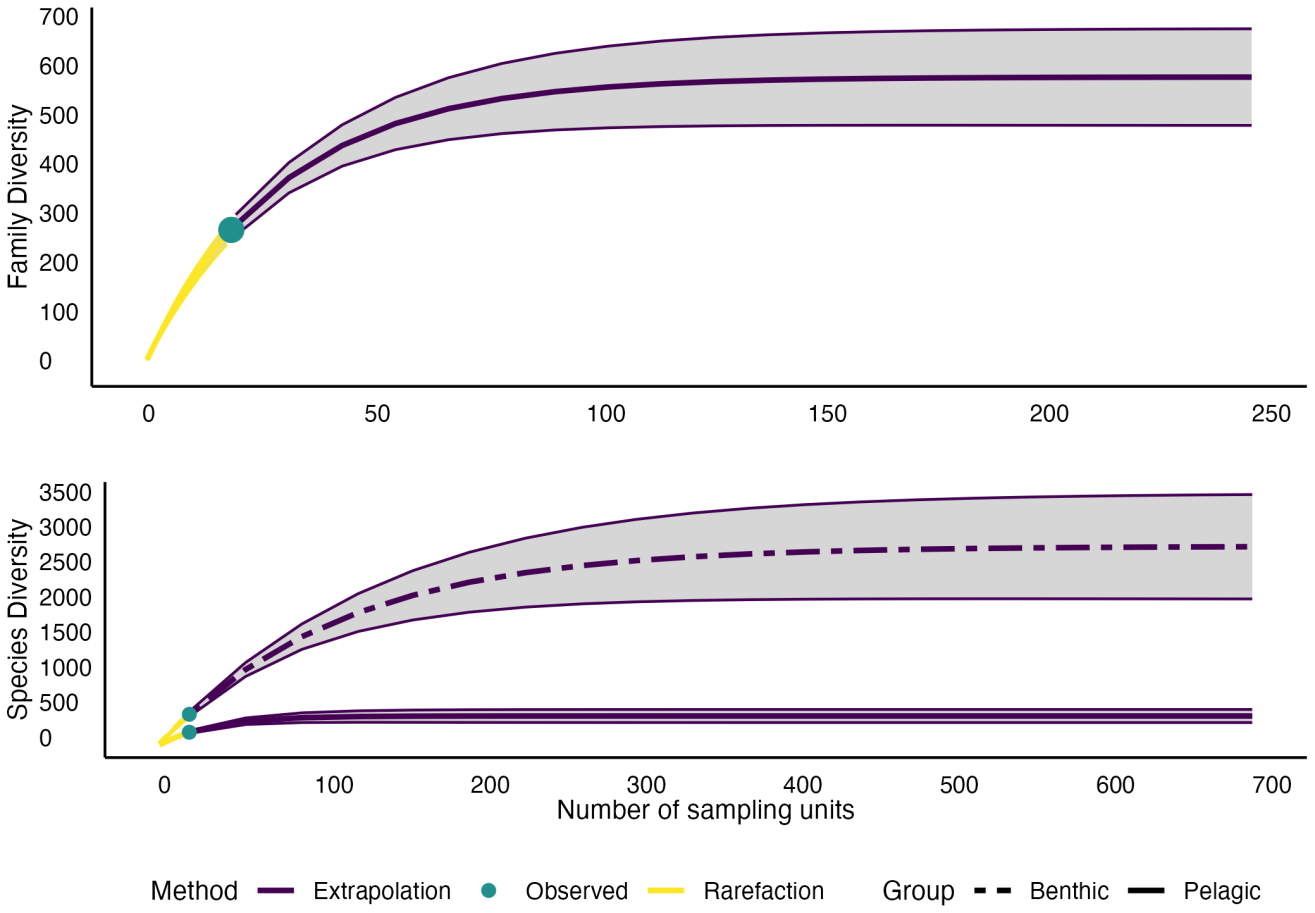
Deep-sea family and species richness within Barbados. (A) 283 families were recorded, with family diversity estimated to reach asymptote at 593 families. (B) 389 benthic species were recorded, with benthic species diversity estimated to reach an asymptote of 2,430 species, and 180 pelagic species were recorded, estimated to reach an asymptote of 387 species.

However, many families and species still remain to be sampled as evidenced by the Chao2 incidence-based estimator (Fig. 7). Extrapolations based on the number of species recorded during each research cruise between the years 1871 and 2012 (449 benthic morphospecies and 193 pelagic morphospecies) indicate that benthic species diversity is estimated to reach an asymptote of 2,430 species (±429), with a 95% confidence interval of 1,588 to 3,270. Pelagic species, with an observed species richness of 193 are estimated to reach asymptote at 387 species (±42), with a 95% confidence interval from 304 to 469 (Fig. 7). Likewise, at the family level, based on the 283 families observed during these expeditions, family diversity is estimated to reach 593 (±63), with a 95% confidence interval of 470 to 717 (Fig. 7). This indicates that approximately only 20% of the species and 48% of the families in Barbados’ deep sea have been recorded.

## Discussion

### A still poorly understood deep sea

This study demonstrates that there is relatively little understanding of the biodiversity that inhabits Barbados’ deep sea, a result of the highly localized and sporadic sampling. Despite a long history of research cruises (1871–2023), sampling has largely been focused on relatively shallow waters, particularly in the southwest region. This is possibly due to the fact that Barbados’ major seaport is located in its capital, Bridgetown, in the same region (Mycoo et al., 2021). Approximately 90% of the records (824 benthic, 597 pelagic) were observed no deeper than 699 metres. As a result, the vast majority of Barbados’ EEZ, where 98% exceeds 1,000 metres depth, remains largely understudied. This pattern echoes findings by Miloslavich et al. (2010) and Allcock et al. (2025), which highlight that despite the expanse of deep water in the Caribbean, research on deep-sea ecosystems has been limited, with very little data collected deeper than 500 metres.

The composition of species observed and the depths they were collected at in Barbados was largely owed to the types of vessels and sampling technologies used, as was seen by Lue Chin et al. (2026) in Trinidad and Tobago. The bulk of the expeditions occurred prior to the 1980s; largely before the methods of studying the deep sea evolved from dredging and trawling to the use of HOVs and ROVs. The prevalence of pelagic trawling likely led to the phylum Chordata accounting for the most records, the majority of which were pelagic species. Gears in the ‘pelagic trawling’ category, such as the 10′ IKMWT, allow for the escape through the meshes of smaller organisms, avoidance by larger, more agile animals, and destruction of fragile organisms, particularly those that are gelatinous (Koslow, Kloser & Williams, 1997). This is clearly demonstrated in the data as the 10’ IKMWT only collected species from the classes Teleostei and Malacostraca, consisting entirely of fishes and shrimp (Table S1). In addition to the indiscriminate nature of dredging and trawling methods, they also collected species in relatively shallower waters, with ROVs and HOVs only collecting in the 4,200–5,600 depth range. ROVs and HOVs are able to selectively sample a much wider range of substrata and species, with ROVs being the only method to record all ten phyla in Barbados. This is because they can employ various tools, such as cameras, corers, suction samplers, mechanical arms, scoops, and specialized nets (Gauthier, Sarrazin & Desbruyères, 2010).

Miloslavich et al. (2010) gathered data from numerous expeditions using methods similar to that of this study, recording twelve phyla within the Caribbean’s deep sea, which included three phyla not found in this assessment (Chaetognatha, Bryozoa, and Brachiopoda). While these phyla were not recorded in our assessment of Barbados’ deep-sea biodiversity, this is a testament that these phyla, among others, possibly remain to be discovered in Barbados’ waters. Overall, the biases witnessed in the dataset hinder attempts to accurately assess the community structure on Barbados’ seafloor or in the water column.

From 1986 onwards, newer technologies such as ROVs and/or HOVs were the primary method of collection and observation, with these being the methods employed at the methane seeps, Volcano A, Atalante East, Manon, Gingersnap, Milano, and Cyclops during RV *Le Nadir* expedition and RV *Atlantis* (AT21-02) expedition (Le Pichon et al., 1990; Van Dover, 2012) (Fig. 1). The exploration of these ecosystems accounts for the majority of the scientific literature published on Barbados’ deep sea (Le Pichon et al., 1990; Vacelet et al., 1996; Olu et al., 1997; Vacelet & Boury-Esnault, 2002; Olu et al., 2010; Toone & Washburn, 2020). Though Le Pichon et al. (1990) briefly described some of the species that exist on these seeps, Olu et al. (1997) was the first ecological study of the methane-seep communities and their distribution, finding that they were predominantly inhabited by vesicomyid bivalves, species of *Lamellibrachia* tubeworms, and clusters of *Cladorhiza methanophila*, along with other organisms (see also Toone & Washburn, 2020). Many of these species rely on the methane and sulphides in the fluids emitted from the seeps, either *via* endosymbiotic relationships with chemoautotrophic bacteria or through direct consumption (Åström et al., 2018; Toone & Washburn, 2020). It would be more than ten years before the RV *Atlantis* in 2012 (AT21-02) collected the third-most samples with the use of ROV *Jason II* in the same region*.* Using a variety of ROV tools, this expedition sampled multiple seep populations for population genetics and demographics, and reared larvae of selected seep invertebrates (Van Dover, 2012). Toone & Washburn (2020) utilised samples provided by this expedition to determine that many species within these communities use chemosynthesis as their method of feeding, with little input from phytodetritus. Despite this, many questions remain on the ecology and connectivity of methane-seep communities, and their links to ecosystem services, highlighting that there is still much to learn about these extensive ecosystems.

The number and distribution of expeditions in Barbados (Fig. 1) has meant that only a handful of seeps (out of likely hundreds that exist in Barbados’ extensive EEZ) have been studied (Brown & Westbrook, 1988). Yet, methane seeps are but one type of deep-sea geomorphic feature in what is a highly varied deep-sea environment. Barbados’ EEZ is also known to host submarine canyons and seamounts. Seamounts provide a dramatic contrast to the surrounding low relief, altering oceanic circulation patterns and inducing local upwellings, which results in seamounts being colonized by a wide range of species, often with a high level of endemism (O’Hara, 2007; Pitcher et al., 2007; Ramirez-Llodra et al., 2011). Equally, submarine canyons, are heterogeneous habitats which accommodate filter feeders such as cnidarians and sponges, and soft-sediment fauna, and can be biodiversity hotspots (Ramirez-Llodra et al., 2011; Vetter, Smith & De Leo, 2010). It is therefore clear, and supported by the species accumulation curves (Fig. 7), that research of these other deep-sea habitats is required for a more comprehensive understanding of Barbados’ deep-sea species richness.

In the quest to undertake more research on Barbados’ deep-sea biodiversity, it is important to consider how the science is conducted and by whom. All of the research vessels identified in this study were affiliated with foreign institutions, primarily from the Global North, which points to a likely prevalence of parachute or colonial science (De Vos, 2022). There was no evidence in the literature of local scientific collaboration during these research cruises, with no co-authorship by local or locally-based researchers. This appears to have been common practice throughout the Caribbean in biodiversity studies up to recent times. For example, 63% of publications on both marine and terrestrial biodiversity between 2000 and 2015 did not include local authors (Vallès et al., 2021). The RV *Atlantis* cruise report, however, does make mention of a St. Lucian student from the Barbados Community College being a part of the expedition; one of seven artists who joined to interpret the deep-sea environment *via* art (BCC (Barbados Community College), 2025). Furthermore, no collected samples appear to have been retained for study or display in local institutions, instead either formally accessioned in public museums or inaccessible in research institutes in the Global North. While this may have been as a result of the parachute practices, it may have also stemmed from a lack of local infrastructure, which could have ultimately been strengthened during those processes (M. Rabone, 2025, pers. comm.).

These colonial research practices have likely contributed to a lack of local deep-sea expertise, and local awareness whether for scientists, decision-makers, policy experts or members of the public (Miloslavich et al., 2010; De Vos, 2022). This low level of local scientific participation may have also resulted in potential missed opportunities for satisfying Barbados’ scientific or management priorities, capacity sharing, bidirectional knowledge exchange, and long-term investment (Miloslavich et al., 2010; Roberts, Downes & Simpson, 2020; De Vos, 2022; Harden-Davies et al., 2022; Spalding et al., 2023). Further, this creates obstacles for Barbados; a country which, under its MSP process, intends to place 30% of its EEZ under protection. As a result, decisions regarding the MSP or multilateral environmental agreements to which Barbados is party to, such as the Convention on Biological Diversity, may be made without adequate data.

### Charting a deep-sea path in Barbados

#### Deep-sea considerations for Barbados

Despite Barbados’ deep sea being a vastly understudied and data-poor environment, it is clear that it hosts rich biodiversity and plays a crucial role in the provision of critical ecosystem services, such as climate regulation (Thurber et al., 2014). For example, climate change is a key topic of national importance, not only because of Barbados’ vulnerability to its numerous impacts, but also as the The Honourable Mia Amor Mottley, Prime Minister of Barbados, has emerged as one of the most prominent voices on this topic globally (Cabot, 2023). Methane seeps, which are present in large numbers in Barbados (Olu et al., 1997; Toone & Washburn, 2020), are known to host organisms that act as important biological filters as they utilize and sequester methane-derived carbon; a greenhouse gas that contributes significantly to climate change (Grupe et al., 2015). Depending on the rate of flow of the seep fluid, chemosynthesis can convert anywhere from 20–80% of methane carbon into biomass and carbonate (Boetius & Wenzhöfer, 2013; Grupe et al., 2015), highlighting the ecosystem’s importance as a carbon sink and providing the important service of regulating the climate (Thurber et al., 2014). It is therefore important that the deep sea’s ability to sequester carbon is not overlooked as a nature-based solution to the climate crisis, warranting further research.

Additionally, globally, methane seeps have been shown to be frequented by commercially harvested species, with seep-derived food sources contributing to the diets of some commercial species (Grupe et al., 2015; Seabrook, De Leo & Thurber, 2019). Grupe et al. (2015) found that densities of species targeted in bottom fishing (*e.g.*, longspine thornyheads, Pacific Dover soles and lithodid crabs) increased in the center and periphery of the Del Mar Seep off California. These examples indicate that the sphere of influence of these ecosystems is larger than initially considered (Grupe et al., 2015; Levin et al., 2016; Seabrook, De Leo & Thurber, 2019), and although unproven thus far, the high densities of chemosynthetic methane-seep and mud-volcano communities in Barbados’ waters may also result in similar benefits. The conservation of these ecosystems in Barbados is therefore important.

Fisheries support is another ecosystem service, which Barbados’ deep sea, beyond the methane seeps, is likely contributing to (Thurber et al., 2014). The fishing industry in Barbados employs over 6,000 persons in harvest and post-harvest, and contributes significantly to the economy, particularly *via* the export of yellowfin tuna (Food and Agricultural Organization, 2020). This species is known to be a frequent user of the deep sea, diving to depths of up to 500 m, though whether it is for foraging, predator avoidance, or anti-parasite behaviour is still under speculation (Dagorn et al., 2006). Additionally, there are likely non-tuna species present in the deep sea, including benthic species that are important components of the food webs of commercially-important species. Further, Barbados may have other deep-sea ecosystems, such as seamounts, that are linked to fisheries (Clark & Koslow, 2007; Clark, Consalvey & Rowden, 2016; Seabrook, De Leo & Thurber, 2019).

These factors emphasise the need for further research, particularly as deep-sea ecosystems are not far removed from the influences of global warming and other anthropogenic-induced changes (Sweetman et al., 2017). The Great Atlantic Sargassum Belt is an example; fueled by an increase in nutrients and shifting ocean and climatic conditions, it has introduced above normal biomass concentrations of *Sargassum* within Eastern Caribbean waters (Méndez-Tejeda & Hernández Ayala, 2026). Evidence shows that a significant portion of surface *Sargassum* eventually reaches the deep sea, often before it has a chance to decompose, however, further studies are warranted to determine its effects on this environment (Baker et al., 2018; Pries et al., 2023).

#### A road map for deep-sea research and management in Barbados

As the Blue Economy accelerates in the deep ocean globally, with associated human impacts, it is critical that governments are equipped with adequate biological baselines to enable informed decision-making. This is especially so in SIDS with EEZs largely comprised of deep ocean, associated with unique governance and management challenges (Ramirez-Llodra et al., 2011; Amon et al., 2022a; D Amon, 2025, pers. comm.). Closing remaining deep-sea scientific gaps will therefore be an essential but monumental task that will require clear direction, substantial resources, and robust coordination and collaboration, with the top priority being strengthened capacity to collect baseline information.

Fortuitously, the Government of Barbados launched its MSP process at the beginning of 2022. Stakeholders, including those in coastal communities, fisheries, civil society, tourism, and the general public, are being engaged to gather information on their use of the ocean in order to develop a comprehensive national plan for management and conservation (Wiggins, 2022; Inter-American Development Bank (IDB), 2024). This has included the definition of environmental goals and objectives, which aim to apply sustainability principles and management practices to reduce threats to marine natural capital and ecosystem services to ensure the protection of the marine environment (Goal 1); promote sustainable and equitable national socio-cultural and economic development through adaptive and responsive use of the ocean space (Goal 2) and; ensure that the MSP process and its outcomes apply good governance principles, practices, and rights-based ethics (Goal 3). Strategic environmental goals and objectives help ensure the protection of deep-sea ecosystems, including through the self-replenishment of populations, prevention of significant losses of genetic diversity, preservation of species richness, communities and habitats, and sustaining vital ecosystem services (Tunnicliffe et al., 2020). Given the importance of ensuring the ongoing MSP process is scientifically informed, as well as the findings of this deep-sea biodiversity assessment, we suggest a potential road map to close existing gaps in scientific research and management regarding Barbados’ deep-sea ecosystems, incorporated into existing steps of the MSP, here.

### Stage 1: synthesise existing deep-sea data and undertake a gap analysis

To facilitate Goals 1 and 2 of the MSP process, the deep-sea biological data synthesised during this study should be integrated with other types of environmental data and knowledge to create a more holistic and comprehensive picture of Barbados’ deep sea. This should include currently inaccessible or proprietary geophysical studies (*e.g.*, from oil and gas companies). This scientific exercise should also be combined with traditional and local ecological knowledge (Tilot et al., 2021; K. Foster, 2026, pers. comm.). This synthesis can provide location-specific knowledge that can enhance the understanding of ecosystem characteristics, including biologically significant areas, vulnerabilities, threat status and protection levels, to inform the MSP, including of culturally significant migratory marine species, the movements and behaviours of commercially-important fishes, and seasonal variability, which may be passed from generation to generation (Johannes, Freeman & Hamilton, 2000; Tilot et al., 2021). This also then allows for a more targeted gap analysis of information that is most needed.

### Stage 2: develop local and regional capacity

There is a need for Barbados, like other SIDS, to increase the capacity to collect, analyse, and use deep-sea data (Amon et al., 2022a; Bell et al., 2023). Deep-sea equipment and research have been largely restricted to more economically developed countries thus far (Aricó et al., 2020; Amon et al., 2022a; Bell et al., 2023). Building partnerships with other nations and entities will facilitate access to additional resources, including education, equipment, expertise, and funding to support deep-sea capacity sharing. Joint research projects and workshops could facilitate knowledge exchange and help to develop best practices, methodologies, and technologies for deep-sea research.

Critically, such partnerships should align with the Alliance of Small Island States’ (AOSIS) Declaration for the Enhancement of Marine Scientific Knowledge, Research Capacity and Transfer of Marine Technology to Small Island Developing States, which calls for equitable partnerships that increase marine scientific knowledge, develop marine research capacity and transfer marine technology in SIDS (Lopes et al., 2026). Equity in such partnerships would allow for joint research agendas, co-developed programs, alignment with local priorities, the building of long-term relationships, and strengthening local capacity (Harden-Davies et al., 2022), which is critical for Barbados to participate meaningfully and eventually lead its own research. It would also ensure there is equal access to data, technology, knowledge, and skills, including the creation of codes of practice that help to ensure that programs meet self-determined needs of relevant parties (Lopes et al., 2026; Harden-Davies et al., 2022). These steps should also be guided by governance systems that ensure that data collection is designed around equity, avoiding dominance from a narrow and powerful minority, and ensuring that the information gathered is shared so it can be used to address threats (Ota et al., 2024).

While local funding opportunities for this research can be explored, including the newly formed Barbados Environmental Sustainability Fund, which was implemented as a result of Barbados’ Debt for Nature Transaction, partnerships can also open other opportunities for support. These could also facilitate transboundary studies and sampling, a practice that is quite limited in the Caribbean (Vallès et al., 2021), but would provide valuable information on ecosystem connectivity and function within the region. Intergovernmental organizations, such as the Caribbean Community (CARICOM), could follow models provided by the Pacific Community (SPC) or Europe’s International Council for the Exploration of the Sea (ICES) to create an organization to coordinate and conduct critical research to inform decisions on the conservation and sustainable use of the marine and coastal environment (Hassanali, 2022). Strengthening capacity in this manner ensures that Goal 3 of the Barbados MSP is reached, particularly in ensuring its longevity and effectiveness.

### Stage 3: create a scientific research agenda and increase baseline knowledge

Given the sporadic nature in which deep-sea research has been conducted in Barbados thus far and following the synthesis of existing environmental data and identification of knowledge gaps, there is a need for more systematic research of the island’s deep-sea ecosystems. To build the baseline environmental information, such as what species exist at which depths and where, a systematic research campaign should be conducted. While there needs to be more spatial diversity in sampling, there also needs to be repeat sampling in the same areas, to begin to elucidate temporal patterns.

While the traditional methods of deep-sea research are expensive and remain out of reach for many SIDS, there are some low-cost technologies that can be employed to gather much-needed information. For example, drift cameras and Baited Remote Underwater Video Systems (BRUVS) are being successfully utilized in SIDS to conduct rapid assessments of deep-sea species and habitats (Dominguez-Carrió, Fontes & Morato, 2021; Giddens et al., 2021; Novy et al., 2022). Not only are these technologies relatively inexpensive when compared to traditional deep-sea technologies, but many are also capable of being deployed from small vessels (Giddens et al., 2021; Dominguez-Carrió, Fontes & Morato, 2021; Novy et al., 2022). Long-term sustainability of such an undertaking would be ensured by integrating it into an existing programme, project or entity (Amon et al., 2022b).

With this in mind, workshops with stakeholders should be hosted to determine a prioritized and detailed research agenda. This would include sampling and monitoring criteria, guided by clear research questions needed to achieve Goal 1 of the MSP, which has objectives of legally declaring 30% of the ocean space as protected biodiversity zones and applying sustainable management to the remaining 70%. This could include specific standardized minimum requirements on what parameters need to be measured, detailed methodologies to be used (including requirements for meeting statistical robustness), and analyses to be carried out, so that data collection, comparison, and synthesis is as efficient as possible in terms of time, effort, and resources in Barbados (Amon et al., 2022b).

### Stage 4: increase awareness of the deep sea

Building Barbados’ deep-ocean awareness is crucial as knowledge of these ecosystems, their importance, and their vulnerability is likely low (Jobstvogt et al., 2014). This can be incorporated in Goal 2 of the MSP, which has objectives of developing curricula and courses to address priority areas; and should include the deep sea. This can include the introduction of more ocean-based modules at a tertiary level, as well as providing scholarships to local talent to pursue studies elsewhere to then return and utilize the transferrable skills gained. Other targeted programs, such as Massive Open Online Courses (MOOCs) on the deep sea, also prove successful in educating large numbers of people from middle to low-income countries, as it can be more accessible than traveling to universities for in-person studies (Fielding, Copley & Mills, 2019).

An increase in awareness and literacy is often hindered by a lack of open sharing of environmental data. Without this, the deep sea will continue to remain an unfamiliar and poorly managed place. Efforts are being made to develop ocean data and information systems, which improve the data availability and enable open-source products and services catered to a broad community of users, including academia and ocean managers (Howell et al., 2020). It is important that these databases are inclusive and equitable and are useful in that the data collected and provided supports efforts to address the climate and biodiversity crises faced globally (Ota et al., 2024). This access to relevant ocean data then better facilitates the efforts of countries in the Global South in their decision-making processes and policy framing (Ota et al., 2024).

Establishing natural history museums is another method of connecting the public with ocean life, through the display of specimens and provision of space for the storage and care of such specimens (Howell et al., 2020). As it stands, the Barbados Museum and Historical Society’s Harewood Gallery hosts a small natural history exhibit, using displays and dioramas. However, no marine samples, and consequently none from the deep sea, are displayed, leaving a gap where the public could otherwise become familiar with the local deep-sea fauna. Given the current lack of local infrastructure, this may be a priority area for increasing capacity and could perhaps extend into the development of a regional network, strengthened by a pooling of resources (M Rabone, 2025, personal communication).

### Stage 5: undertake regular synthesis and review

The Government of Barbados, under the MSP Unit, should conduct five-year reviews to determine what has been done, its effectiveness, and what remains to be completed to ensure that scientific methodologies, monitoring and management remain adequate and reflect the best available science (Amon et al., 2022b). Van den Burg et al. (2023) identify five main analyses that need to be undertaken when monitoring and evaluating MSPs: physical conditions, transformed spatial structures, demographic structure, social structure, and social and economic conditions. This therefore enables the Government of Barbados to optimize its role as a regulator, effectively designate and refine area-based management tools, and develop and refine environmental management targets and thresholds as needed (Amon et al., 2022b). Challenges can arise, however, as significant datasets are needed, and often, sufficient resources are not dedicated to the monitoring and evaluation processes (Santos et al., 2021; Van den Burg et al., 2023). This is why ensuring that monitoring and evaluation are readily measurable and cost-effective is critical (Douvere & Ehler, 2011).

## Conclusion

Barbados’ deep-sea biodiversity remains largely unknown and poorly understood. However, results show that despite the limited, highly localized, and sporadic sampling thus far, substantial biodiversity exists. As the effects of climate change continue to worsen, and Barbados makes plans for its ocean space through its MSP process, this study provides a concise understanding of Barbados’ known deep-sea biodiversity, providing a starting point from which the island can begin to bridge existing scientific gaps. A key step will be further systematic sampling and study. The majority of samples and observations occurred close to land and on the seafloor, and when these activities moved offshore, samples were largely pelagic. Waters deeper than 700 m had comparatively less sampling, meaning that in an EEZ where 99% of the area is deeper than 1,000 m, there is a significant lack of information. With richness extrapolations indicating that potentially 80% of species and 53% of families remain to be discovered, it is clear much more study is required. Key to closing the gaps will be synthesising known biodiversity information with other environmental datasets and knowledge types, strengthening local capacity to conduct its own deep-sea research and undertaking regular reviews of this information. Increasing deep-ocean literacy among the population is also a key component in closing gaps, as understanding the deep ocean’s unseen influence and how it is changing will aid in its preservation.

## Supplemental Information

10.7717/peerj.21380/supp-1 Supplemental Information 1 Barbados’ Deep-Sea Biological Records.

10.7717/peerj.21380/supp-2 Supplemental Information 2 Barbados Deep-Sea Species List.

## Additional Information and Declarations

### Data Availability

The following information was supplied regarding data availability: The raw data is available at Zenodo: - Palacios-Abrantes, J. (2026). Repository for analysis on paper on Barbados deep-sea biodiversity. Zenodo. https://doi.org/10.5281/zenodo.19361030
